# Prescription opioid use and misuse among adolescents and young adults in the United States: A national survey study

**DOI:** 10.1371/journal.pmed.1002922

**Published:** 2019-11-05

**Authors:** Joel D. Hudgins, John J. Porter, Michael C. Monuteaux, Florence T. Bourgeois

**Affiliations:** 1 Division of Emergency Medicine, Boston Children’s Hospital, Boston, Massachusetts, United States of America; 2 Departments of Pediatrics and Emergency Medicine, Harvard Medical School, Boston, Massachusetts, United States of America; 3 Pediatric Therapeutics and Regulatory Science Initiative, Computational Health Informatics Program, Boston Children’s Hospital, Boston, Massachusetts, United States of America; Massachusetts General Hospital, UNITED STATES

## Abstract

**Background:**

Prescription opioid misuse has become a leading cause of unintentional injury and death among adolescents and young adults in the United States. However, there is limited information on how adolescents and young adults obtain prescription opioids. There are also inadequate recent data on the prevalence of additional drug abuse among those misusing prescription opioids. In this study, we evaluated past-year prevalence of prescription opioid use and misuse, sources of prescription opioids, and additional substance use among adolescents and young adults.

**Methods and findings:**

This was a retrospective analysis of the National Survey on Drug Use and Health (NSDUH) for the years 2015 and 2016. Prevalence of opioid use, misuse, use disorder, and additional substance use were calculated with 95% confidence intervals (CIs), stratified by age group and other demographic variables. Sources of prescription opioids were determined for respondents reporting opioid misuse. We calculated past-year prevalence of opioid use and misuse with or without use disorder, sources of prescription opioids, and prevalence of additional substance use. We included 27,857 adolescents (12–17 years of age) and 28,213 young adults (18–25 years of age) in our analyses, corresponding to 119.3 million individuals in the extrapolated national population. There were 15,143 respondents (27.5% [95% CI 27.0–28.0], corresponding to 32.8 million individuals) who used prescription opioids in the previous year, including 21.0% (95% CI 20.4–21.6) of adolescents and 32.2% (95% CI 31.4–33.0) of young adults. Significantly more females than males reported using any prescription opioid (30.3% versus 24.8%, *P* < 0.001), and non-Hispanic whites and blacks were more likely to have had any opioid use compared to Hispanics (28.9%, 28.1%, and 25.8%, respectively; *P* < 0.001). Opioid misuse was reported by 1,050 adolescents (3.8%; 95% CI 3.5–4.0) and 2,207 young adults (7.8%; 95% CI 7.3–8.2; *P* < 0.001). Male respondents using opioids were more likely to have opioid misuse without use disorder compared with females (23.2% versus 15.8%, respectively; *P* < 0.001), with similar prevalence by race/ethnicity. Among those misusing opioids, 55.7% obtained them from friends or relatives, 25.4% from the healthcare system, and 18.9% through other means. Obtaining opioids free from friends or relatives was the most common source for both adolescents (33.5%) and young adults (41.4%). Those with opioid misuse reported high prevalence of prior cocaine (35.5%), hallucinogen (49.4%), heroin (8.7%), and inhalant (30.4%) use. In addition, at least half had used tobacco (55.5%), alcohol (66.9%), or cannabis (49.9%) in the past month. Potential limitations of the study are that we cannot exclude selection bias in the study design or socially desirable reporting among participants, and that longitudinal data are not available for long-term follow-up of individuals.

**Conclusions:**

Results from this study suggest that the prevalence of prescription opioid use among adolescents and young adults in the US is high despite known risks for future opioid and other drug use disorders. Reported prescription opioid misuse is common among adolescents and young adults and often associated with additional substance abuse, underscoring the importance of drug and alcohol screening programs in this population. Prevention and treatment efforts should take into account that greater than half of youths misusing prescription opioids obtain these medications through friends and relatives.

## Abstract 2

Joel Hudgins and coworkers study reported use and misuse of prescription opioids and other substances among adolescents and young adults in the United States.

## Author summary

**Why was this study done?:**

- Prescription opioid misuse is a leading cause of unintentional injury and death among adolescents and young adults.
- There is limited information on the source of prescription opioids among adolescents and young adults or whether those misusing prescription opioids engage in use of additional substances and drugs of abuse.
- Understanding these factors will inform strategies to ensure judicious opioid prescribing and effective treatment approaches.

**What did the researchers do and find?:**

- Using the National Survey on Drug Use and Health for the years 2015 and 2016, we determined past-year prevalence of prescription opioid use, sources of prescription opioids, and additional substance use among adolescents and young adults ages 12–25.
- We found that 27.5% of respondents, corresponding to an estimated 32.8 million individuals, used prescription opioids in the previous year, including 21.0% of adolescents and 32.2% of young adults.
- The prevalence of opioid misuse was 3.8% among adolescents and 7.8% among young adults.
- Most individuals misusing prescription opioids obtained them for free from a friend or relative or from a single prescriber.
- Individuals with prescription opioid misuse reported high prevalence of use of other substances, including cocaine, hallucinogens, heroin, and inhalants.

**What do these findings mean?:**

- The prevalence of prescription opioid use is high among adolescents and young adults in the United States despite known risks for progression to opioid and other substance use disorders in this population.
- Prevention and treatment efforts should take into account that adolescents and young adults misusing prescription opioids obtain these drugs most commonly from friends and relatives or from a single prescriber.
- Healthcare providers should consider screening all adolescents and young adults with opioid misuse for additional substance use and should have established intervention plans available to maximize the opportunity to provide substance use treatment to this population.

## Introduction

Over the past two decades, opioid misuse and poisonings have emerged as a public health crisis in the US. Since 1999, rates of deaths secondary to opioids have tripled, and in 2017 alone, over 72,000 Americans died from opioid overdoses [1,2]. Children and adolescents have not been spared, with substantial increases over the past two decades in opioid-related emergency department visits, hospital and intensive care unit admissions, and deaths [3–6]. Opioid exposures accounted for over 12% of all deaths in 2016 among 15- to 24-year-olds, which represents a 4-fold increase since 2001 [7]. According to the Centers for Disease Control’s 2018 National Vital Statistics report, unintentional injuries are now the leading cause of death among adolescents and young adults, with poisonings the most common unintentional injury [8,9].

Among adults in the US, roughly 1 in 3 is estimated to use prescription opioids, with 4.7%, or 11.5 million, misusing them [10]. Opioid misuse in adults has been linked to several risk factors, including mood and anxiety disorders, male gender, educational attainment, and age at first misuse [11–13]. Among adolescents and young adults, data are sparser and less consistent, although prevalence of opioid use disorder appears to be steadily increasing [14,15]. A recent meta-analysis examining past-year prevalence of prescription opioid misuse among adolescents and young adults reported estimates ranging from 0.7% to 16.3% [16]. Two of the largest adolescent drug surveys in the US are the Youth Risk Behavior Survey, which asks about misuse of “pain medications” broadly, and Monitoring the Future, which asks about misuse of “narcotics other than heroin” [17,18]. These studies report lifetime prevalence of misuse of 17.0% and 6.8%, respectively, among 12th grade students in 2017. Risk factors identified among adolescents include earlier onset of use, educational attainment, and chronic pain, although these links are less robust than in adult studies [19–21]. Importantly, legitimate use of opioids during adolescence appears to predispose to later opioid misuse [19,22].

There is limited information on how adolescents and young adults are obtaining prescription opioids. Studies suggest an indirect link between opioid prescriptions in adults and exposures in adolescents, suggesting that households and family members may be a contributing source [23,24]. In one survey study conducted from 2008 to 2011, parents and friends from school were identified as the most common sources of prescription opioids for adolescents [25].

In this study, we analyzed data from a large, nationally representative survey collecting information on prescription opioids to measure prevalence of opioid use and misuse among US adolescents and young adults. We also determined sources of prescription opioids and characterized opioid use and misuse according to additional use of other substances and drugs of abuse.

## Methods

### Survey methods

Data for this analysis were obtained from survey responses in the 2015 and 2016 National Survey on Drug Use and Health (NSDUH). The NSDUH is an annual cross-sectional survey that collects information on drug use, mental health, and other health-related issues in the US civilian, noninstitutionalized population aged 12 years and older. It is sponsored by the Substance Abuse and Mental Health Services Administration (SAMHSA) within the US Department of Health and Human Services. We used the publicly available version of the dataset, which was downloaded for use on February 16, 2018.

The survey includes interviews with approximately 70,000 individuals randomly selected annually, using a multistage area probability sample for each state and the District of Columbia. Certain populations, including younger age groups (i.e., 12 to 25 years), are oversampled to ensure robust estimates [26]. Interviews are conducted in the individual’s residence by a trained interviewer after verbal informed consent has been obtained. Data are collected using computer-assisted personal interviewing, in which the interviewer reads a question to the participant and enters the response into the computer. For questions on illicit drug use and other sensitive behaviors, a more private approach is used with audio computer-assisted self-interviewing, enabling respondents to read or listen to a question on headphones and enter the response into the computer themselves [26]. Respondents receive a $30 compensation for their participation. Additional information on the NSDUH sample design and survey methodology is available in the SAMHSA annual reports [26].

Our analysis was prospectively planned, including the definition of the study population, sociodemographic characteristics of interest, and outcome measures. Two modifications were made to the planned analysis. The first was our definition of young adult, which we initially defined as 18–23 years of age but modified to 18–25 years to match the NSDUH definition and data categorization. The second was the grouping of the different sources of prescription opioids, which was revised in response to peer-review comments. The study was deemed exempt from review by the Institutional Review Board at Boston Children’s Hospital.

### Study population

We analyzed responses from adolescents 12 to 17 years of age and young adults 18 to 25 years of age. The NSDUH seeks to allocate 25% of its sample to adolescents aged 12 to 17 years, 25% to young adults aged 18 to 25 years, and the remaining 50% to adults 26 years and older [26]. The actual percentages of adolescents and young adults participating in 2015 were 23.1% and 24.6%, respectively, and in 2016, 23.3% and 23.9%, respectively [26]. The weighted interview response rates for adolescents and young adults were 77.7% and 74.4%, respectively, in 2015, and 77.0% and 72.3%, respectively, in 2016. By comparison, weighted interview response rates for adults were 67.4% in 2015 and 66.7% in 2016. All respondents were sampled independently. During the study period, less than 1% of respondents in our population were re-sampled in successive years. Each sampled observation is weighted within the context of the sampling frame for the given year, regardless of whether the observation represents a repeated record on the same individual.

### Outcome measures

We examined three outcomes based on survey questions in the NSDUH: prescription opioid use, misuse, and use disorder, sources of prescription opioids for misuse, and use of other substances and drugs of abuse. NSDUH specifically identifies prescription opioids by presenting a list of prescription opioids and pictures of prescription opioid pills to respondents, and asks about past month, year or lifetime use of these opioids. For participants providing a positive response, follow-up questions determine classification into one of three groups: use without misuse, misuse without use disorder, or use disorder. Prescription opioid misuse is defined as “use in any way that a doctor did not direct you to use them” [27]. Specific examples constituting misuse are provided, including use without a prescription and using prescription opioids “more than you intended to” [28]. Opioid use disorder is classified as “recurrent use which causes clinically significant impairment, including health problems, disability, and failure to meet major responsibilities at work, school, or home,” based on *Diagnostic and Statistical Manual of Mental Disorders*, 4th edition (DSM-IV), criteria for substance use disorder [29,30].

For respondents reporting misuse of prescription opioids in the past year, NSDUH collects data on where the medications were obtained. Respondents are asked to select as many responses as apply from the following options: (a) obtained from one doctor, (b) obtained from more than one doctor, (c) stole from doctor office, clinic, hospital, or pharmacy, (d) obtained from friend or relative for free, (e) bought from friend or relative, (f) stole from friend or relative, (g) bought from drug dealer or stranger, and (h) got some other way.

The NSDUH also asks respondents about additional substance use and drug abuse. For our population, we examined responses to questions on tobacco, alcohol, cocaine, cannabis, heroin, inhalant, and hallucinogen use. Questions address use within the past month, past year, and lifetime. We combined use with and without use disorder for these substances and drugs.

Sociodemographic characteristics collected in the NSDUH include sex, race/ethnicity (non-Hispanic white, non-Hispanic black, Hispanic, and Asian/Native American), health insurance (private, Medicare, Medicaid, uninsured, or other), marital status, educational attainment, and past-year family income (<$20,000, $20,000–$49,999, $50,000–$74,999, and ≥$75,000).

The reliability of the NSDUH questionnaire related to substance use and misuse has been previously assessed, with measures showing good reproducibility over time [31,32]. Missing values in NSDUH are imputed by SAMHSA prior to release of the dataset using a predictive mean neighborhood (PMN) and a modified PMN as detailed in the NSDUH codebook [26]. PMN is a method of imputing missing or ambiguous values that is similar to predictive mean matching and has been used in the survey since 1999 [33]. The median percentage of values imputed for the initial demographic variables was 3.5% in 2015 and 3.7% in 2016. For the prescription drug variables, the median percentage of imputed values was 1.0% in 2015 and 0.65% in 2016 [26,34].

### Statistical analysis

Data were analyzed using the person-level sample weight, which is the product of the corresponding sampling fractions at each stage in the sample design, and allows extrapolation to national population estimates. The sampling weights are adjusted using the generalized exponential model [35], which adjusts for survey nonresponse, post-stratification, and extreme weights, yielding an unbiased national estimate of occurrences and characteristics. When generating population estimates from survey data, we accounted for the survey design by specifying the primary sampling units and the degrees of freedom provided by NSDUH to ensure accurate estimates [34]. All analyses were performed in STATA Version 15 (StataCorp, College Station, TX) using the suite of estimation commands for survey data (*svyset* and *svy*).

We calculated descriptive statistics for adolescents and young adults with past-year use of prescription opioids, both overall and stratified by misuse and use disorder. We also assessed the sources of prescription opioids, overall and stratified by misuse and use disorder, and calculated the prevalence of additional use of other substances and drugs of abuse, stratified by prescription opioid use and misuse. Results were calculated and reported with 95% confidence intervals (CIs), and *P* values for comparisons were calculated using chi-squared test. The study conforms to the STROBE guideline (S1 STROBE Checklist).

## Results

The NSDUH included 27,857 adolescent and 28,213 young adult respondents during the survey years 2015 to 2016, corresponding to an estimated 119.3 million individuals in the extrapolated national population (49.8 million adolescents and 69.5 million young adults). Overall, 27.5% of these respondents, corresponding to an estimated 32.8 million individuals, reported using a prescription opioid in the previous year, including 21.0% (95% CI 20.4–21.6) of adolescents and 32.2% (95% CI 31.4–33.0) of young adults (Table 1).

**Table 1:** Demographic characteristics of adolescents and young adults with past-year prescription opioid use, misuse, and use disorder^a^.

**Characteristic**	**Any Use of Prescription Opioid (*n* = 15,143)^b^**	**Prescription Opioid Use without Misuse (*n* = 11,886)**	**Prescription Opioid Misuse without Use Disorder (*n* = 2,801)**	**Prescription Opioid Use Disorder (*n* = 456)**
	**Percent (95% CI)**	**Percent (95% CI)**	**Percent (95% CI)**	**Percent (95% CI)**
**Age**				
12–17	21.0 (20.4–21.6)	82.1 (80.7–83.5)	15.2 (13.9–16.5)	2.7 (2.1–3.3)
18–25	32.2 (31.4–33.0)	75.9 (74.7–77.1)	21.1 (19.8–22.3)	3.0 (2.5–3.5)
**Sex**				
Male	24.8 (24.2–25.4)	73.7 (72.0–75.3)	23.2 (21.6–24.8)	3.1 (2.5–3.7)
Female	30.3 (29.7–30.9)	81.4 (80.3–82.4)	15.8 (14.7–16.9)	2.8 (2.4–3.2)
**Race/ethnicity**				
Non-Hispanic white	28.9 (28.2–29.6)	76.6 (75.5–77.8)	20.2 (18.9–21.4)	3.2 (2.7–3.7)
Non-Hispanic black	28.1 (27.1–29.2)	80.5 (78.7–82.4)	17.6 (15.6–19.4)	1.9 (1.1–2.7)
Hispanic	25.8 (24.7–26.9)	78.8 (76.7–80.8)	18.2 (16.3–20.1)	3.0 (2.4–3.6)
Non-Hispanic other	22.8 (21.1–24.4)	79.6 (76.1–83.1)	17.8 (14.5–21.2)	2.6 (1.6–3.5)
**Health Insurance**				
Private only	26.9 (26.1–27.6)	78.2 (76.9–79.5)	19.2 (17.8–20.6)	2.6 (2.1–3.0)
Uninsured	28.8 (27.3–30.4)	73.1 (69.6–76.6)	23.2 (20.0–26.4)	3.7 (2.2–5.2)
Medicaid only	27.8 (27.0–28.7)	79.1 (77.6–80.6)	17.4 (15.8–19.0)	3.5 (2.8–4.1)
Other	29.4 (27.3–31.5)	77.9 (74.9–80.9)	19.8 (16.9–22.7)	2.3 (1.3–3.2)
**Family income**				
<$20,000	29.4 (28.5–30.4)	76.7 (74.8–78.6)	20.8 (18.8–22.7)	2.5 (2.0–3.0)
$20,000– $49,999	28.6 (27.9–29.3)	77.1 (75.6–78.6)	19.7 (18.4–20.9)	3.2 (2.5–3.9)
$50,000– $74,999	27.2 (25.8–28.6)	79.7 (77.5–81.9)	16.7 (14.6–18.9)	3.6 (2.5–4.7)
>$75,000	25.2 (24.1–26.2)	78.8 (77.1–80.5)	18.5 (16.7–20.3)	2.7 (2.1–3.3)
**Metropolitan statistical area**				
Large	26.4 (25.9–26.9)	78.6 (77.2–80.0)	18.6 (17.2–20.0)	2.8 (2.2–3.3)
Small	28.3 (27.3–29.3)	77.2 (75.3–79.1)	20.0 (18.3–21.7)	2.8 (2.2–3.5)
Nonmetropolitan	30.2 (29.2–31.1)	76.7 (74.4–79.1)	19.6 (17.5–21.7)	3.7 (2.7–4.6)

^a^Values are weighted percentages and 95% CIs.

^b^The extrapolated population estimates for the different opioid use categories are 32.8 million individuals with any prescription opioid use, 25.6 million with prescription opioid use without misuse, 6.3 million with prescription opioid misuse without use disorder, and 1 million with prescription opioid use disorder.

Abbreviation: CI, confidence interval

Opioid misuse or use disorder in the past year was reported by 6.1% (95% CI 5.8–6.4) of respondents, corresponding to an estimated 1.9 million adolescents (3.8%; 95% CI 3.5–4.0) and 5.4 million young adults (7.8%; 95% CI 7.3–8.2; *P* < 0.001). Prevalence of opioid misuse without use disorder was higher in young adults compared with adolescents (21.1% [95% CI 19.8–22.3] versus 15.2% [95% CI 13.9–16.5], respectively; *P* < 0.001), with similar prevalence of opioid use disorder in the two groups. Considering both age groups together, female respondents were more likely to have had any prescription opioid use compared with males (30.3% [95% CI 29.7–30.9] versus 24.8% [95% CI 24.2–25.4], *P* < 0.001), but male respondents were more likely to have opioid misuse without use disorder compared with females (23.2% [95% CI 21.6–24.8] versus 15.8% [95% CI 14.7–16.9], respectively; *P* < 0.001). Non-Hispanic white and black respondents reported higher prevalence of past-year opioid use compared with Hispanic respondents (*P* < 0.001), but prevalence of misuse without and with use disorder were similar between groups. Insurance status was not a significant determinant of opioid use, but uninsured respondents were more likely to report opioid misuse without use disorder compared with those with Medicaid.

Among those with prescription opioid misuse, 25.4% (95% CI 23.5–27.2) obtained them from the healthcare system, 55.7% (95% CI 53.7–57.6) from friends or relatives, and 18.9% (95% CI 17.4–20.5) through other means (Table 2). Adolescents with opioid misuse obtained opioids most commonly for free from a friend or relative (33.5%; 95% CI 28.7–38.3) or by prescription from a single doctor (19.2%; 95% CI 16.4–22.1). Far fewer obtained them by stealing from a healthcare facility (1.7%; 95% CI 0.5–2.8), through prescriptions from multiple doctors (2.2%; 95% CI 1.3–3.2), or by buying them from a drug dealer or stranger (6.5%; 95% CI 4.4–8.6). Sources of opioids for young adults with opioid misuse were similar to adolescents, with free procurement from friends or relatives (41.4%; 95% CI 38.8–44.1) and prescription from a single doctor (24.0%; 95% CI 22.1–25.9) the most common sources.

**Table 2:** Extrapolated population estimates of source of prescription opioids among adolescents and young adults with opioid misuse^a^.

Source of Opioid	Any Opioid Misuse (*n* = 3,257)^b^			
	Adolescents (*n* = 1,050)		Young adults (*n* = 2,207)	
	*n*	Percent (95% CI)	*n*	Percent (95% CI)
**Obtained from healthcare system**	433,462	23.1 (20.0–26.3)	1,407,643	26.1 (24.1–28.1)
One doctor	359,795	19.2 (16.4–22.1)	1,293,571	24.0 (22.1–25.9)
More than one doctor	42,051	2.2 (1.3–3.2)	71,562	1.3 (0.6–2.0)
Stole from doctor’s office, clinic, hospital, or pharmacy	31,616	1.7 (0.5–2.8)	42,510	0.8 (0.3–1.3)
**Obtained from friends or relatives**	920,296	49.2 (44.8–53.6)	3,124,468	57.9 (55.4–60.5)
Obtained from friend or relative for free	626,662	33.5 (28.7–38.3)	2,233,560	41.4 (38.8–44.1)
Bought from friend or relative	151,486	8.1 (6.2–10.0)	657,733	12.2 (10.4–14.0)
Took from friend or relative without asking	142,148	7.6 (5.5–9.7)	233,175	4.3 (3.2–5.4)
**Obtained from other source**	516,681	27.6 (23.9–31.3)	859,474	15.9 (14.1–17.7)
Bought from drug dealer or stranger	121,009	6.5 (4.4–8.6)	422,616	7.8 (6.3–9.4)
Other	159,678	8.5 (6.8–10.3)	235,532	4.4 (3.4–5.3)
Unknown	235,994	12.6 (9.7–15.6)	201,326	3.7 (2.9–4.6)

^a^Values are weighted percentages and 95% CIs.

^b^The extrapolated population estimate is 7.2 million, of which 1.9 million are adolescents and 5.4 million young adults.

Abbreviation: CI, confidence interval

Additional substance use and drug abuse associated with prescription opioid use is shown in Table 3. Among adolescents and young adults with prescription opioid use without misuse, 50.5% (95% CI 49.2–51.7) had previously used tobacco, 70.5% (95% CI 69.3–71.7) had used alcohol, and 43.9% (95% CI 42.7–45.1) had used marijuana. These prevalence rates were significantly higher for respondents with opioid misuse, with 78.4% (95% CI 76.7–80.1; *P* < 0.001) reporting use of tobacco, 90.1% (95% CI 89.0–91.3; *P* < 0.001) use of alcohol, and 80.7% (95% CI 79.2–82.2; *P* < 0.001) use of marijuana. For cocaine, heroin, hallucinogen, and inhalant use, differences in prevalence of use between those with opioid use without misuse and those with opioid misuse were even more pronounced. Prevalence of cocaine use increased greater than 4-fold from 7.9% (95% CI 7.1–8.7) to 35.5% (95% CI 33.1–38.0; *P* < 0.001) for respondents with opioid use without misuse compared with those with opioid misuse. Similarly, the prevalence in these populations increased from 0.9% (95% CI 0.6–1.1) to 8.7% (95% CI 7.1–10.2; *P* < 0.001) for heroin use, 13.1% (95% CI 12.3–13.9) to 49.4% (95% CI 46.9–51.8; *P* < 0.001) for hallucinogen use, and 12.1% (95% CI 11.3–12.8) to 30.4% (95% CI 28.1–32.8; *P* < 0.001) for inhalant use.

**Table 3:** Additional substance use and drug abuse among adolescents and young adults with prescription opioid use^a^.

Type of Substance Use	Any Use of Prescription Opioid (*n* = 15,143)^b^	Prescription Opioid Use without Misuse (*n* = 11,886)	Prescription Opioid Misuse with and without Use Disorder (*n* = 3,257)
	Percent (95% CI)	Percent (95% CI)	Percent (95% CI)
**Tobacco use**			
Past month used	32.2 (31.1–33.2)	25.5 (24.5–26.5)	55.5 (53.4–57.6)
Ever used	56.6 (55.5–57.8)	50.5 (49.2–51.7)	78.4 (76.7–80.1)
Never used	43.4 (42.2–44.5)	49.5 (48.3–50.8)	21.6 (19.9–23.3)
**Alcohol use**			
Past month used	49.2 (48.2–50.2)	44.2 (42.9–45.4)	66.9 (64.8–69.1)
Ever used	74.9 (73.9–75.8)	70.5 (69.3–71.7)	90.1 (89.0–91.3)
Never used	25.1 (24.2–26.1)	29.5 (28.3–30.7)	9.8 (8.7–11.0)
**Cannabis use**			
Past month used	23.2 (22.2–(24.3)	15.7 (14.7–16.7)	49.9 (47.9–51.8)
Ever used	52.1 (51.0–53.1)	43.9 (42.7–45.1)	80.7 (79.2–82.2)
Never used	47.9 (46.9–49.0)	56.1 (54.9–57.3)	19.3 (17.8–20.8)
**Cocaine use**			
Past month used	2.0 (1.6–2.4)	0.8 (0.5–1.1)	6.2 (4.9–7.5)
Ever used	14.0 (13.1–14.9)	7.9 (7.1–8.7)	35.5 (33.1–38.0)
Never used	86.0 (85.1–86.9)	92.1 (91.3–92.9)	64.5 (62.0–66.9)
**Heroin use**			
Past month used	0.4 (0.3–0.5)	—^c^	1.7 (1.2–2.2)
Ever used	2.6 (2.2–3.0)	0.9 (0.6–1.1)	8.7 (7.1–10.2)
Never used	97.4 (97.0–97.8)	99.1 (98.9–99.4)	91.3 (89.8–92.9)
**Hallucinogen use**			
Past month used	2.6 (2.2–2.9)	0.9 (0.7–1.1)	8.2 (6.9–9.5)
Ever used	21.2 (20.2–22.1)	13.1 (12.3–13.9)	49.4 (46.9–51.8)
Never used	78.8 (77.9–79.8)	86.9 (86.1–87.7)	50.6 (48.2–53.1)
**Inhalant use**			
Past month used	0.9 (0.7–1.1)	0.6 (0.4–0.8)	2.2 (1.4–2.9)
Ever used	16.1 (15.5–16.8)	12.1 (11.3–12.8)	30.4 (28.1–32.8)
Never used	83.9 (83.2–84.5)	87.9 (87.2–88.7)	69.5 (67.2–71.9)

^a^Values are weighted percentages and 95% CIs.

^b^The extrapolated population estimates for the different opioid use categories are 32.8 million individuals with any prescription opioid use, 25.6 million with prescription opioid use without misuse, and 7.2 million individuals with prescription opioid misuse with and without use disorder.

^c^Too few respondents to provide a reliable value.

Abbreviation: CI, confidence interval

Among adolescents and young adults with opioid misuse, prevalence of additional substance use was significantly higher among young adults for all substances except inhalant use (Table 4). These differences were seen both for past month use and for any prior use of substances. Among adolescents, past month use of tobacco, alcohol, and cannabis was 31.2% (95% CI 28.4–34.1), 36.7% (95% CI 32.9–40.5), and 35.3% (95% CI 32.5–38.0), respectively. These prevalence rates increased to 63.9% (95% CI 61.3–66.5; *P* < 0.001), 77.4% (95% CI 75.3–79.5; *P* < 0.001), and 55.0 (95% CI 52.4–57.5; *P* < 0.001), respectively, among young adults. For cocaine and hallucinogens, lifetime prevalence of using the substance more than doubled between adolescents (11.5% [95% CI 9.3–13.7] and 25.4% [95% CI 22.2–28.6], respectively) and young adults (43.9% [95% CI 40.8–47.0], *P* < 0.001; and 57.7% [95% CI, 54.7–60.7], *P* < 0.001, respectively) with opioid misuse.

**Table 4:** Additional substance use and drug abuse stratified by age group for adolescents and young adults who misuse prescription opioids^a^.

Type of Substance Use	Any Opioid Misuse (*n* = 3,257)^b^			
	Adolescents (*n* = 1,050)		Young adults (*n* = 2,207)	
	*N*	Percent (95% CI)	*N*	Percent (95% CI)
**Tobacco use**				
Past month used	583,845	31.2 (28.4–34.1)	3,446,446	63.9 (61.3–66.5)
Ever used	1,006,791	53.8 (50.3–57.3)	4,685,714	86.9 (85.2–88.6)
Never used	863,649	46.2 (42.7–49.7)	705,872	13.1 (11.4–14.8)
**Alcohol use**				
Past month used	685,825	36.7 (32.9–40.5)	4,173,481	77.4 (75.3–79.5)
Ever used	1,344,868	71.9 (68.7–75.1)	5,201,606	96.5 (95.6–97.4)
Never used	525,571	28.1 (24.9–31.3)	189,980	3.5 (2.6–4.4)
**Cannabis use**				
Past month used	659,497	35.3 (32.5–38.0)	2,963,319	55.0 (52.4–57.5)
Ever used	1,049,843	56.1 (52.0–60.3)	4,809,436	89.2 (87.9–90.4)
Never used	820,597	43.9 (39.7–48.0)	582,150	10.8 (9.6–12.0)
**Cocaine use**				
Past month used	53,586	2.9 (1.6–4.1)	396,461	7.4 (5.6–9.1)
Ever used	214,878	11.5 (9.3–13.7)	2,366,733	43.9 (40.8–47.0)
Never used	1,655,561	88.5 (86.3–90.7)	3,024,853	56.1 (53.0–59.2)
**Heroin use**				
Past month used	—^c^	—^c^	118,289	2.2 (1.5–2.9)
Ever used	—^c^	—^c^	608,090	11.3 (9.2–13.4)
Never used	1,849,545	98.9 (98.3–99.4)	4,783,496	88.7 (86.6–90.8)
**Hallucinogen use**				
Past month used	99,775	5.3 (3.7–6.9)	497,457	9.2 (7.6–10.9)
Ever used	475,271	25.4 (22.2–28.6)	3,110,102	57.7 (54.7–60.7)
Never used	1,395,168	75.6 (71.4–77.8)	2,281,484	42.3 (39.3–45.3)
**Inhalant use**				
Past month used	57,223	3.1 (1.9–4.7)	100,134	1.8 (1.0–2.7)
Ever used	558,956	29.9 (26.1–33.7)	1,652,648	30.7 (27.7–33.6)
Never used	1,311,484	70.1 (66.3–73.9)	3,738,938	69.3 (66.4–72.3)

^a^Values are weighted percentages and 95% CIs.

^b^The extrapolated population estimate is 7.2 million, of which 1.9 million are adolescents and 5.4 million young adults.

^c^Too few respondents to provide a reliable value.

Abbreviations: CI, confidence interval

## Discussion

In this national sample of adolescents and young adults, 27.5% reported using a prescription opioid in the past year, with 3.8% of adolescents and 7.8% of young adults engaged in opioid misuse or having a use disorder. Respondents stated that opioids were obtained most frequently from friends or relatives or from a single prescriber, and much less often through drug dealers or from multiple prescribers. Adolescents and young adults with any type of opioid misuse were significantly more likely to use additional substances and drugs of abuse, with a third or more reporting prior use of cocaine, hallucinogens, and inhalants and half or more reporting past month use of tobacco, alcohol, or cannabis. Overall, young adults engaging in opioid misuse have higher rates of additional substance use and drug abuse compared with adolescents.

These findings are consistent with prior research indicating that the opioid epidemic is taking a significant toll on adolescents and young adults. In one large longitudinal study of youths 13 to 25 years of age, new diagnoses of opioid use disorder increased 6-fold between 2001 and 2014 [14]. Exposures to opioids have also been accompanied by rising rates of opioid poisonings and overdoses among adolescents and young adults. For adolescents 15 to 19 years of age, the annual rate of hospitalizations for opioid poisonings has increased by greater than 170%, while opioid-related deaths have increased by roughly 250% since the late 1990s [5,15]. Overall, among adolescents and young adults, overdose deaths reached an all-time high of 12.6 per 100,000 in 2017, with the majority of these overdoses linked to opioids [36].

The risk of progression to opioid use disorder and other substance abuse is well-documented for youths exposed to prescription opioids [22,37–39]. High school seniors receiving a first-time medical prescription for an opioid have been shown to have a 33% increased risk of future opioid misuse after high school [19]. This risk increases for adolescents engaging in nonmedical use of prescription opioids. In one survey study, among adolescents engaging in even occasional (3–9 lifetime uses) nonmedical prescription opioid use, greater than 50% met criteria for a substance use disorder by age 35 [38]. In addition, prescription opioid use among adolescents and young adults is linked to progression to heroin use. An analysis of NSDUH data from 2004 to 2011 showed that the hazard of heroin initiation was 13 times higher among adolescents and young adults 12 to 21 years of age with a history of nonmedical prescription opioid use compared with those without such prior use [40]. Overall, 76% of respondents who reported a history of heroin use had previously engaged in nonmedical use of prescription opioids. This risk of progression from nonmedical prescription opioid use to heroin use appears to be significantly greater for young adults compared with persons 25 years and older [41].

Sources of prescription opioids have not been well defined among adolescents and young adults misusing these drugs. Adults misusing prescription opioids have been found to obtain opioids both from prescribers and through friends and relatives [10]. Previous work among middle and high school students indicates that diversion of opioids, defined as selling, trading, giving away, or loaning prescription opioids, plays an important role in opioid misuse, with as many as 22% of students with medical use of prescription opioids approached to divert their opioid medication [42,43]. Our findings confirm these reports and quantify sources of prescription opioids, indicating that nearly half of adolescents and 58% of young adults misusing opioids receive them from friends and relatives. In addition, 22% of adolescents and 25% of young adults obtain opioid medications from prescribers, pointing to another area for targeted intervention for reducing opioid misuse. However, it should be noted that very few respondents received prescriptions from multiple prescribers, indicating that prescription drug monitoring programs—which are designed to monitor opioid prescriptions across providers and settings—alone may be insufficient as a policy approach to reduce opioid misuse in youths.

Adolescents and young adults misusing opioids are at high risk of abusing other substances. In addition to our findings of high prevalence of recent substance abuse, prior studies have demonstrated high prevalence of explicit co-ingestion of drugs with prescription opioids. Among high school seniors, approximately 70% report co-ingesting another drug while engaging in prescription opioid misuse, with greater than half reporting concurrent use of marijuana or alcohol, and 10% concurrent use of cocaine, tranquilizers, or amphetamines [44]. These patterns highlight the importance of screening adolescents and young adults with opioid misuse for use of other substances. In the emergency department setting, screening followed by brief interventions have been shown to be well received and effective at reducing alcohol and marijuana use among adolescents and young adults [45–47]. Providers should consider screening all adolescents and young adults presenting to the emergency department or other healthcare settings with opioid misuse for additional substance use. Healthcare facilities should also have established intervention plans or referral options available to maximize the opportunity to provide substance use treatment to this population.

Our study has several limitations. While the NSDUH had a high response rate of approximately 75% during the years analyzed, we cannot preclude self-selection bias among participants. In addition, despite sophisticated interviewing techniques, we cannot exclude socially desirable reporting among participants. The NSDUH also does not survey homeless people, active military personnel, or people in prison, which might impact findings reported for young adults in the study, especially because high rates of opioid misuse and use disorder have been identified in these populations [48,49]. Because the study does not employ a longitudinal design, we were unable to evaluate patterns of opioid use or progression to other substance use and drug abuse over time for individuals.

## Conclusions

Our findings suggest that the prevalence of prescription opioid use among adolescents and young adults in the US is high despite known risks for future opioid and other drug use disorders. Prevention and treatment efforts should take into account that greater than half of adolescents and young adults misusing prescription opioids obtain these drugs from friends and relatives. Both adolescents and young adults misusing opioids demonstrate high prevalence of additional substance use and drug abuse, underscoring the importance of drug and alcohol screening programs in this population.

## Supporting information

S1 STROBE Checklist STROBE statement—A checklist of items that should be included in reports of observational studies. (DOC)

## Abbreviations

CI: confidence interval
DSM-IV: *Diagnostic and Statistical Manual of Mental Disorders*, 4th edition
NSDUH: National Survey on Drug Use and Health
PMN: predictive mean neighborhood
SAMHSA: Substance Abuse and Mental Health Services Administration
